# Challenges to hypertension and diabetes management in rural Uganda: a qualitative study with patients, village health team members, and health care professionals

**DOI:** 10.1186/s12939-019-0934-1

**Published:** 2019-02-28

**Authors:** Haeyoon Chang, Nicola L. Hawley, Robert Kalyesubula, Trishul Siddharthan, William Checkley, Felix Knauf, Tracy L. Rabin

**Affiliations:** 1 Department of Epidemiology (Chronic Disease), Yale University School of Public Health, New Haven, CT USA; 2 African Community Center for Social Sustainability (ACCESS), Nakaseke, Uganda; 3 Department of Physiology, Makerere University College of Health Sciences, Kampala, Uganda; 4 Division of Pulmonary and Critical Care, School of Medicine, Johns Hopkins University, Baltimore, MD USA; 5 Center for Global Noncommunicable Disease Training and Research, Johns Hopkins University, Baltimore, MD USA; 6 Department of Nephrology and Medical Intensive Care, Charité – Universitätsmedizin Berlin, Berlin, Germany; 7 Department of Internal Medicine, Yale University School of Medicine, New Haven, CT USA; 8 Uganda Initiative for Integrated Management of Non-Communicable Diseases, Kampala, Uganda

**Keywords:** Hypertension, Diabetes, Uganda, Rural health, Chronic diseases, Qualitative, Village health team

## Abstract

**Background:**

The prevalence of hypertension and diabetes are expected to increase in sub-Saharan Africa over the next decade. Some studies have documented that lifestyle factors and lack of awareness are directly influencing the control of these diseases. Yet, few studies have attempted to understand the barriers to control of these conditions in rural settings. The main objective of this study was to understand the challenges to hypertension and diabetes care in rural Uganda.

**Methods:**

We conducted semi-structured interviews with 24 patients with hypertension and/or diabetes, 11 health care professionals (HCPs), and 12 community health workers (known as village health team members [VHTs]) in Nakaseke District, Uganda. Data were coded using NVivo software and analyzed using a thematic approach.

**Results:**

The results replicated several findings from other settings, and identified some previously undocumented challenges including patients’ knowledge gaps regarding the preventable aspects of HTN and DM, patients’ mistrust in the Ugandan health care system rather than in individual HCPs, and skepticism from both HCPs and patients regarding a potential role for VHTs in HTN and DM management.

**Conclusions:**

In order to improve hypertension and diabetes management in this setting, we recommend taking actions to help patients to understand NCDs as preventable, for HCPs and patients to advocate together for health system reform regarding medication accessibility, and for promoting education, screening, and monitoring activities to be conducted on a community level in collaboration with village health team members.

## Introduction

Non-communicable disease (NCD) related deaths are expected to increase globally [1]. One third of NCD mortalities globally have been attributed to cardiovascular diseases (CVD) [2, 3]. About 80% of the CVD-related deaths occur in low and middle-income (LMIC) countries, and more than half of these deaths are a result of complications of hypertension (HTN) and diabetes (DM) [3, 4]. In Sub-Saharan Africa (SSA) the burden of disease is expected to double for both HTN and DM by 2025 and 2035 respectively [5, 6]. The increase in prevalence of DM is especially rapid in Uganda, where the number of people with DM is predicted to increase by 166.9% between 2013 and 2035, outpacing most other countries [5, 7].

Poor control of HTN and DM under the direct influence of lifestyle factors and lack of awareness in Uganda is the major contributor to the increase in these conditions [8–10]. A nationwide NCD risk factor survey conducted in 2015 concluded that awareness is more problematic in Uganda than earlier smaller studies suggest and that lack of awareness disproportionately affects rural compared to urban communities [9, 11–13]. The survey found that only 6.0% of patients with HTN in rural areas were aware of their condition, as opposed to 12.1% of patients with HTN in urban areas [14]. Another NCD risk factor survey conducted in 2016 in selected peri-urban and rural areas of Uganda found 95.6% of those identified to have DM in rural areas were unaware of their condition while far fewer (65.0%) were unaware of their health status in urban areas [11]. Adding to this disparity and the challenge to management of these conditions, the 2018 Service Availability and Readiness Assessment survey conducted in Uganda found that availability of HTN and DM medications were limited to health facilities located in urban areas [15].

While several studies have now described the challenges associated with HTN and DM care in urban areas of Uganda, less is known about the hurdles in rural settings [16]. Our study addresses this gap and aims to understand the challenges to HTN and DM care in rural Uganda using qualitative research methods. We collected and analyzed interviews from three key stakeholders in rural Nakaseke District: health care professionals (HCP), community health workers (known locally as village health team members [VHTs]), and patients with hypertension and/or diabetes. We also make recommendations based on the findings to improve HTN and DM care in rural Uganda.

## Methods

Semi-structured face-to-face in-depth interviews were conducted with HCPs, VHTs, and patients in order to identify challenges to HTN and DM care in rural Uganda from the viewpoints of the three key stakeholder groups.

### Setting

Nakaseke is a rural district in Central Uganda approximately 66 km north of Kampala with an estimated population of 191,100 [17]. It consists of nine administrative units or counties including Kasangombe, Nakaseke sub-county, and Nakaseke Town Council. These three counties were chosen to maximize geographic variability in patient care resources. The patients and VHTs each lived in one of these three sub-counties and the HCPs were recruited from public and private hospitals in these three counties.

### Participants and sampling

Adult individuals with HTN and/or DM who were participants in the *Nakaseke Community - Based Cohort for Non-Communicable Diseases: Community Based Models of Prevention, Care and Management for Non-Communicable Diseases (NCD Study)* [Mulago Hospital Research and Ethics Committee (MREC) #1079] cohort study were recruited to participate in thissub-study. The *NCD Study* was a collaboration between investigators at Yale, Charité Berlin, Johns Hopkins, and Makerere Universities, as well as ACCESS (African Community Center for Social Sustainability), a local non-governmental organization (NGO) and includes 16,694 adults aged over 40 years living in Nakaseke District, Uganda for at least two years prior to study initiation, who have previously been evaluated for chronic respiratory disease, HTN, DM, and kidney disease. An initial list of VHTs working in Nakeseke District was obtained from ACCESS, and additional VHTs were found through VHT interviewee referrals (snowball sampling). The HCPs were recruited initially based on referrals from the administration at Nakeseke Hospital (Ministry of Health district hospital), and additional HCPs were recruited using a snowball sampling approach as well.

**Fig. 1:**
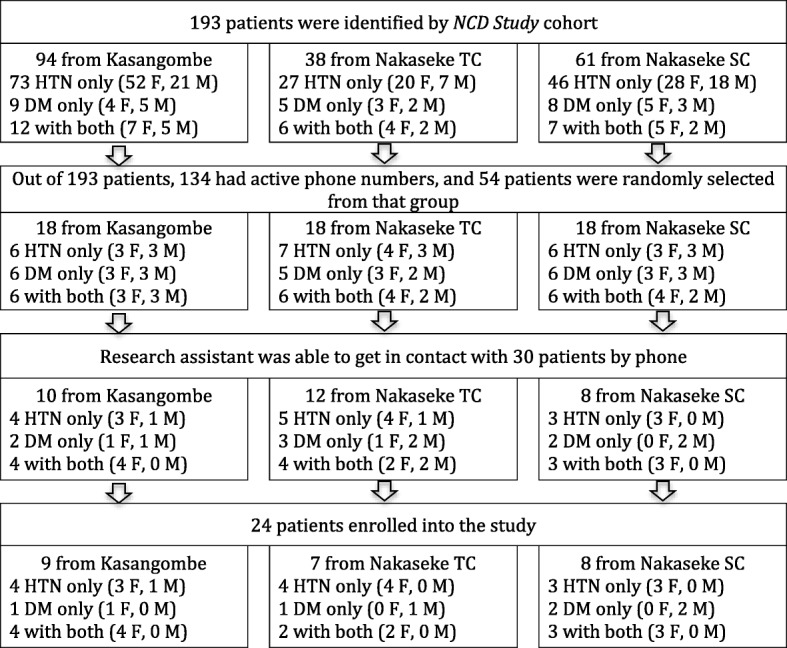
Flowchart of patient recruitment. *TC = Town Council; SC = Sub-county*

To maximize variation among patients, we purposely selected three axes of diversity using a theoretical sampling method: *gender, the chronic condition (HTN and/or DM),* and the *participant’s county of residence (Kasangombe, Nakaseke sub-County, or Nakaseke Town Council).* Gender was chosen as an axis based on the potential for differences between men and women in their health seeking behaviors. The patients’ chronic condition determined their experience with the illness and with the process of HTN and/or DM management. The last axis was participant county of residence. Nakaseke Town Council is the location of the Ministry of Health’s district hospital, Nakaseke Hospital. Residents of Nakeseke Town Council have higher income and better access to resources with a greater number of shops and amenities available. Nakaseke sub-county is the next closest county to the Nakaseke Hospital, and Kasangombe is located the farthest away. Residents of both Nakaseke sub-county and Kasangombe, have lower incomes and limited access to amenities compared to Nakeseke Town Council. The geographic variation in the availability of resources and distance to Nakaseke Hospital may impact participants’ experiences with access to health care. To ensure geographic representation, an equal number of patients were selected from each of the three counties (Fig. 1 *Flow chart of patient recruitment*). For similar reasons, VHTs were selected using their gender and counties in which they served as the axes of diversity. For HCPs, their position in the health system was used as the major axis of diversity.

Participants with HTN and/or DM were eligible to participate if they satisfied all the following requirements: a) age ≥ 18 years, b) lived in Nakaseke District for at least two years, c) were capable of understanding study procedures and providing informed consent, and d) had received treatment for HTN and/or DM for at least one full year at health care facilities in Nakaseke (to be able to adequately speak to their experience). HCPs were eligible to participate if they satisfied all of the following requirements: a) involvement in the care and treatment of patients with HTN or DM, b) practiced location within Nakaseke District, and c) employment as an HCP in Nakaseke District for one year or more. VHTs were eligible to participate if they satisfied all the following requirements: a) active involvement in the care and treatment of individuals in Nakaseke District and, b) worked in Nakaseke District for one year or more. We recruited a total of 24 patients with HTN and/or DM, 11 HCPs, and 12 VHTs.

### Data collection

A trained research assistant contacted participants from the NCD study by phone. Patient participants had given their consent to be contacted for additional research. All participants were asked screening questions to confirm their eligibility for the interview, invited to participate, and, if interested, scheduled for the interview. During the research visit, which took place at the patient homesteads or place of work (HCPs and VHTs), the research assistant obtained verbal informed consent from the participants because the study posed minimal risk of harm to participants, and the research context did not require written consents out of respect for cultural concerns regarding signed consent documents. Interviews were conducted in Luganda for patients and VHTs, and in English for HCPs.

Semi-structured face-to-face in-depth interviews of 30 to 60 min were conducted either by the first author, by the research assistant or both together. The interview guide consisted of open-ended questions with prompts for probes (interview guides for patients and HCPs are shown in Table 1). Questions were asked about the process of diagnosis and treatment of hypertension and diabetes, ways in which patients engaged in self-management of hypertension and diabetes, barriers and facilitators to hypertension and diabetes treatment, and the current and possible roles of VHTs in hypertension and diabetes management. All interviews were audio-recorded with hand-written field notes. All participant interviews and VHT interviews in Luganda were transcribed and then translated into English by the research assistant, who is fluent in both languages. The interviews conducted in English were transcribed by either the first author or research assistant.

**Table 1:** Semi-Structured Interview Guides for Patient and HCP interviews*

**Domain I. Introductory Question on HTN and DM and associated care**	
Patient	
1. Can you describe to me your typical day? Probe: How does HTN/DM play a role in your daily routine? 2. Can you tell me how and when you found out you have HTN/DM? Probe: What was that experience like for you? Knowledge and Attitudes 3. If you had to explain to a family member what HTN/DM is, how would you explain it? Probe: What do you think caused your HTN/DM? Do you think HTN/DM is preventable? Why or why not? Do you think HTN/DM is curable? Why or why not? 4. What have you done in the past and what are you currently doing to manage HTN/DM? Probe: Can you tell me what medications or other remedies you are taking or had taken? Does diet play a role? If so how? Does exercise play a role? If so how?	
HCP	
1. Can you describe what your role is in this healthcare facility? 2. What happens to a patient with HTN/DM who comes to this facility? Can you walk me through that process?	
**Domain II. HTN and DM care support and care system**	
Patient	
5. Who do you go for treatment when you feel sick? Probe: Do you go to traditional healers or other non-medical providers? If yes, can you tell me more about it? Who do you trust to give you advice or information about HTN/DM? 6. Do you feel comfortable sharing your diagnosis of HTN/DM with your family members? Why or why not? 7. Would you feel comfortable sharing your diagnosis of HTN/DM with your neighbors or community? Why or why not? 8. What resources are available in your community for learning about HTN/DM?	
HCP	
3. How are HTN/DM diagnosed in this community? 4. How do people with HTN/DM manage and treat their disease in this community? 5. Can you tell me about any specific guidelines followed by HCPs in Nakaseke district for HTN/DM diagnosis and management? 6. What can patients with HTN/DM do to be actively involved in their health management? 7. Tell me about any recommendations you give to your patients for managing HTN/DM? Probe: What are the characteristics of patients that are more likely to listen to your advice and recommendations? 8. Have there been efforts to improve the care of patients with HTN/DM in this community and facility? 9. Tell me about any community-based programs or resources available to adults with HTN/DM and their caregivers/family members in Nakaseke district?	
**Domain III. Barriers and Facilitators**	
Patient	
9. Tell me about the difficult parts of HTN/DM treatment access and drug adherence for adults with HTN and DM in Nakaseke. 10. Tell me about the facilitators of HTN/DM treatment access and drug adherence for adults with HTN and DM in Nakaseke. 11. How satisfied are you with the health care services you receive for HTN and DM? Please describe.	
HCP	
10. Tell me about the barriers to HTN/DM treatment access and adhrerence for adults with HTN/DM in Nakaseke? Probe: Tell me what challenges you face in diagnosing HTN/DM. What barriers do you face in following these guidelines? 11. What common challenges do you face in educating patients about their HTN and DM diagnosis and/or management? Probe: Are there common knowledge gaps, myths, or misconceptions that patients have that contribute to this? 12. How does the use of traditional and/or complementary and alternative medicine play a role in the lives of your patients with HTN/DM? Probe: How does it play a role in their decision to seek healthcare in general? And in their decision to seek care for HTN/DM? Are there specific characteristics of patients who are more likely to seek care from traditional healers or other non-medical providers? 13. Tell me about the facilitators of HTN/DM treatment access and adherence for adults with HTN/DM in Nakaseke?	
**Domain IV. The goal is to help participants discuss the role of VHTs.**	
Patient	
12. Have you interacted with or received care from a VHT? 13. Do you believe VHTs are an important part of the health system? 14. Do you think that VHTs could play a role in helping you manage your HTN and DM?	
HCP	
14. What is the role of VHTs in the management of patients with HTN/DM? Probe: How do you perceive the quality of care delivered by VHTs?	
**Ending Questions**	
Patient	
15. What do you wish you knew more about, related to HTN/DM? 16. How would you like to receive information about HTN/DM and how to manage it?	
HCP	
15. How comfortable do you feel managing patients with HTN/DM? Please describe. Probe: *(If any answer other than comfortable)* what would help you feel more comfortable in managing patients with HTN/DM? 16. Can you think of any other HCPs with HTN/DM related training in the community that would be useful for us to speak with?	

*The guide used for VHT interviews are not included

### Analysis

For analysis, we used both inductive and deductive approach to thematic analyses [18] where multiple experiences and perspectives contribute to developing the final research outcome. The first and second authors (HC, NLH) collaborated to create the initial code book for each group of stakeholders. Using the initial code book, two coders (HC, NJ) coded 24 patient transcripts, and two coders (HC, MR) coded 12 VHT transcripts and 11 HCP transcripts using Microsoft Word. The first author (HC) and the coders (NJ, MR) adopted line by line coding to capture emerging themes using a mix of descriptive and conceptual codes and refined the codebooks through weekly discussions. Once the codebook was finalized based on consensus, the same coders coded the remaining patient, VHT, and HCP interviews. Once all transcripts were coded and consensus reached among coders, the first author (HC) compiled the portions of coded texts and exported into QSR NVivo software version 11. The first author (HC) reviewed the quotes with themes and concepts on challenges to HTN and DM care and drafted a preliminary analysis. The analysis was then refined through discussions with the research team.

### Ethical approval and consent

Ethical approval for this study was granted by the Yale University Human Subjects Committee, Mulago Hospital Research and Ethics Committee (MREC) and the Uganda National Council of Science and Technology (Reference numbers 2000021365; MREC 1079, SS 4283 respectively). All participants provided verbal consent to participate in the study and agreed to anonymized data from their interviews being published.

## Results

**Fig. 2:**
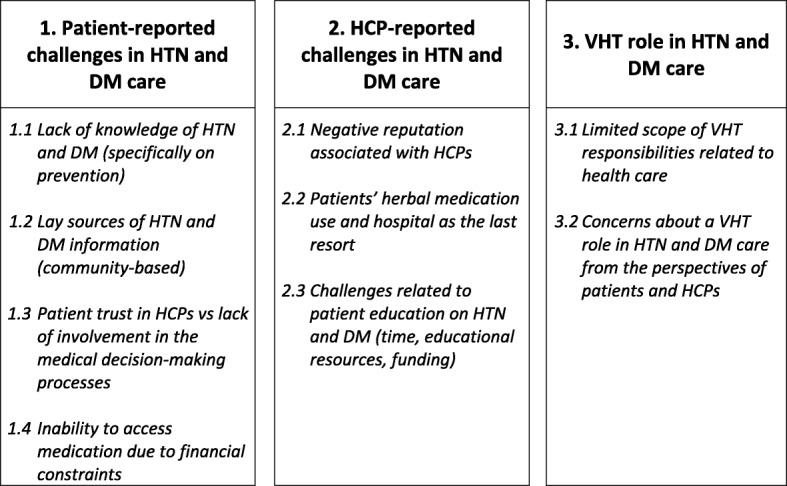
Summary of major themes and sub-themes

The thematic analysis produced three major themes and 9 sub-themes from interviews with 24 patients with HTN and/or DM, 11 HCPs, and 12 VHTs. Figure 2 provides an overview of the relationship between the major themes and the sub-themes (Fig. 2 *Summary of major themes and sub-themes*). Table 2 provides demographic characteristics of the 47 total participants. Tables 3, 4 and 5 summarize the major themes and additional participant quotes related to challenges in HTN and DM care, identified from interviewing the three key stakeholder groups. Participants’ quotes are contextualized by characteristics such as the chronic condition (HTN and/or DM), gender, the participant’s county of residence, and the participant identification number.

**Table 2:** Characteristics of Patients, HCPs, and VHTs who participated in the study

Characteristic	Patients (*N* = 24)	HCPs (*N* = 11)	VHTs (*N* = 12)
Age group^a^			
18–30	0	1 (9.1%)	0
31–40	0	3 (27.3%)	4 (33.3%)
41–50	5 (22.7%)	3 (27.3%)	3 (25.0%)
51–60	10 (45.5%)	4 (36.4%)	5 (41.7%)
61–70	3 (13.6%)	0	0
71–80	2 (9.1%)	0	0
81–90	2 (9.1%)	0	0
Sex			
Female	20 (83.3%)	8 (72.7%)	6 (50.0%)
Male	4 (16.7%)	3 (27.3%)	6 (50.0%)
Education level^b^			
None	1 (4.2%)	0	0
Primary	12 (50.0%)	0	5 (41.7%)
Secondary	5 (20.8%)	2 (18.2%)	6 (50.0%)
Post-secondary (Not including certificate)	2 (8.3%)	9 (81.8%)	1 (8.3%)
Diagnosis		N/A	N/A
Hypertension only	11 (45.8%)		
Diabetes only	4 (16.7%)		
Hypertension and diabetes concurrently	9 (37.5%)		
Village			
Kasangombe	9 (37.5%)	1 (9.1%)	4 (33.3%)
Nakaseke sub county	8 (33.3%)	0	4 (33.3%)
Nakaseke town council	7 (29.2%)	10 (90.9%)	4 (33.3%)
HCP role	N/A		N/A
Nurse		4 (36.4%)	
Doctor		3 (27.3%)	
Pharmacist		2 (18.2%)	
Nutritionist		1 (9.1%)	
Private Pharmacist		1 (9.1%)	

The percentages might not add up to 100% due to rounding

^a^Missing two participants from the group of patients

^b^Missing four participants from the group of patients

**Table 3:** Themes defining patient challenges in HTN and DM care

**Lack of knowledge of HTN and DM**	
*They are not communicable like HIV […] You can’t prevent hypertension or diabetes. (HTN and DM, Female, Kasangombe, P4)* **I don’t know. No [I can’t even speculate the cause], because I never used to take sugar, indeed I was so perplexed when I was told that I am diabetic. I couldn’t understand […] I didn’t have any pain, or anything that would prompt me to go to the hospital, I was just there feeling nothing. (DM only, Male, Nakaseke sub-county, P10)** *If someone shares about HIV what about diabetes, I wouldn’t hide it even from a woman because she cannot contract diabetes, diabetes is not communicable. (DM only, Male, Nakaseke town council, P9)* **Yes [diet has many roles in managing diabetes]. It helps because there are times when I get blurred vision when I delay eating food, and when you eat and take medication too it is well for you. Food moves with the drugs it helps to transport blood. (DM only, Male, Nakaseke town council, P9)** *You might have palpitations and also get worried of children’s school fees, then you find increased blood sugar level but you are on drugs. So the doctor asks, “are you taking your medication?” “Yes doctor I do take,” “what are you worried about” and you come to realize that there is something distressing you. (HTN and DM, Female, Nakaseke sub-county, P6)* **You hear them speak of what shouldn’t be eaten but we have not yet gotten a teaching about how to conduct our lives. (DM only, Female, Kasangombe, P11)**	
**Lay sources of HTN and DM information**	
*Yes, [I feel comfortable sharing with family members and neighbors] because somebody might advise and recommend you to a better medication or to traditional medicines. (HTN only, Female, Nakaseke Sub-county, P15)* **I hear food with starch should not be eaten like cassava. [I get this information] from our colleagues. (DM only, Female, Kasangombe, P11)** *Why wouldn’t I share with them [friends and neighbors]? [I share with them because] maybe they could give me some advice. (HTN only, Female, Kasangombe, P23)* **My friend [advises me on hypertension]. They [my friends] also have hypertension. (HTN only, Female, Nakaseke town council, P12)** *There is no problem [sharing with neighbors about the condition] because I explain to many of my friends, and relatives who say that they get heat in the body, sweating, thirst because I also get these symptoms. I tell them to reduce on taking sugar, reduce on eating fatty foods. (DM only, Male, Nakaseke sub-county, P10)* **I feel comfortable. Because I want to help them be more aware of hypertension. I try to tell them to avoid getting worried when they get a similar problem because worrying precedes hypertension. (HTN only, Female, Kasangombe, P20)**	
**Patient trust in HCPs** **vs** **lack of involvement in the medical decision-making processes**	
*Their [the prescribed drugs] names! They [the doctors] just give me the drugs, I don’t know the names. I am just given the drugs. (HTN and DM, Female, Kasangombe, P4)* **I know them [the drugs], they are dispensed to me. […]do I know how to read your English [to name the drugs]? (HTN and DM, Female, Nakaseke town council, P1)** *I am not a doctor, unless I bring you the prescription book but I don’t know the drugs. (HTN and DM, Female, Kasangombe, P5)* **Sometimes we find that the drugs are finished, and we are told to go and buy from places were the drugs are, at times you don’t have money and you have to first work to make the money then go and buy the drugs. (DM only, Female, Kasangombe, P11)** *When I take it, I feel like throwing up, I feel disgusted in my heart. [when this happens,] I first take some break. I take a break for about 2 days such that these that I have taken descend down (DM only, Female, Kasangombe, P11)* **I buy [drugs], they are not there at the hospital that is the problem. […] The government has not endeavored to know that diabetes exists it the country and that it affects people, I thought that I was the first to ever get this disease and I almost became hopeless but I realized that we were about 680 patients […] but there are those who die without ever going to the hospital. (DM only, Male, Nakaseke sub-county, P10)** *“We are in a good relationship with the doctors, because they comfort us and teach us what we need to do to live.” (DM only, Male, Nakaseke sub-county, P8)* **“It is doctors [I trust to give information on HTN and DM]. We get our blood sugar level measured […] and if it has increased then I resume taking drugs. Sometimes I take drugs, sometimes I don’t, so you take one tablet once a day for hypertension and for diabetes one tablet in the morning and one in the evening.” (HTN and DM, Female, Nakaseke town council, P3)** *That [information on any diseases] comes from a doctor and nobody can diagnose a disease it is only a doctor who can do that [provide information]. (DM only, Male, Nakaseke sub-county, P8)* **“Many times, the person to trust [on HTN and DM information] is the one who also has the disease. The second is the health worker. Not the traditional herbalists, but the real professional health worker because they are well trained.” (HTN and DM, Female, Nakaseke sub-county, P18)**	
**Inability to access medication due to financial constraints**	
*“I was told to started on drugs, and to regularly come to [the hospital to] pick up the drugs. However sometimes I go there and the drugs are not available. I also don’t have money. Then I keep quiet with nothing to do.”* *(HTN only, Female, Nakaseke town council, P17)* **The only problem is when there are no drugs in the hospital. When the drugs are finished, it is you who has to look for them to survive. (DM only, Male, Nakaseke sub-county, P9)** *Problem is lack of money to buy capsules to take. Even to get the drugs from Nakaseke is far and, boda boda [motorcycle] are also expensive. When there are no free drugs in hospitals, all you get is Panadol. (HTN only, Female, Nakaseke sub-county, P16)* **You have to take your prescription. [It is easy to get drugs] If it is available [in the hospital] but right now I need money to buy the drugs. ** **(HTN only, Female, Kasangombe, P23)** *I had not encountered any [difficulties] yet because we have been getting the government drugs and even when there are only a few, at least they divide and everyone gets a little and we are informed to come back [to the hospital] when that is finished. We get to pick more drugs if the stocks are refilled. (HTN and DM, Female, Nakaseke sub-county, P7)*

**Table 4:** Themes defining HCP challenges in HTN and DM care

**Negative reputation associated with HCPs**	
*Some people fear our attitudes, they don’t want to come to the hospital. (Female, Nurse at Nakaseke Hospital, HCP 1)* **They [patients] can fear the health workers […] the way you handle that patient can affect the patient to come back to the hospital. […] If we are friendly with the patients it will be ok. (Female, Nurse at Nakaseke Hospital, HCP 1)** *When [patients] reach the hospital, we tell them “you have high levels” then they get worried. […] [We tell the patients] Once a diabetic is always a diabetic. So that worries also. It will take long to make one stabilize [to manage their condition].* *(Female, Nurse at Nakaseke Hospital, HCP 8)* **Some [patients] say some staffs are harsh. Some say, if you’re going to hospital, people might think they have HIV. So, most people fear hospitals. […] When a patient has taken drugs for 1 year then starts taking herbals, herbals fail, it [symptoms of DM] comes back, and someone [a HCP] could abuse her [the patient], “why weren’t you on diabetic drugs? Now you’re coming back?” such things. You shout at her. It doesn’t feel good. (Female, Pharmacist at Nakaseke Hospital, HCP 5)**	
**Patients’ herbal medication use and hospital as the last resort**	
*People believe in herbal so much even they may not come [to the hospital]. Person comes in too late and say that we’ve been using herbal. […] hospital is always the last solution. (Female, Nurse in Nakaseke Hospital, HCP 3)* **Sometimes even those patients can take [traditional medicine] and sometimes they don’t come to the hospital. But at the end, they end up coming back here because they have tried that traditional herbs and it doesn’t work. (Female, Nurse at Nakaseke Hospital, HCP 1)** *Especially those [patients] ones in the village, they are not educated very well. “I was thinking herbal drugs,” those are the stories they give us. […] They come in with their conditions worse.* *(Female, Nurse at Nakaseke Hospital, HCP 3)* **Some [patients] don’t have enough drugs because at times they come here [hospital] and drugs are out of stock, [which is] another reason they feel that these herbals are better than tablets. (Female, Doctor at Nakaseke Hospital, HCP 7)**	
**Challenges related to patient education on HTN and DM**	
*We’ve not done enough of the counseling, because we don’t have the time […] Diabetes and hypertension needs time [in counseling] because, you need to talk about lifestyles. For both hypertension and diabetes, when we talk about weight reduction, it needs a lot of skills, it needs a lot of examples, needs a lot of talking. (Male, Doctor in Kasangombe, HCP 9)* **If we have budget for some fuel and go to villages, you can health educate these people, you talk about symptoms of hypertension and diabetes, you can health educate the community. (Female, Nurse at Nakaseke Hospital, HCP 1)** *We do our best to convince them [the patients] and tell them the complications [of medication non-adherence]. But it is a struggle to convince them to take their hypertensive drugs. If I take the blood pressure, [and I] say it is ok, they [patients] don’t take their drugs.* *(Female, Nurse at Nakaseke Hospital, HCP 3)* **They [patients] are good at forgetting. They easily forget so we continue to health educate them. (Female, Nurse at Nakaseke Hospital, HCP 2)**

**Table 5:** Themes defining VHT roles in HTN and DM care

**Limited scope of VHT responsibilities related to health care**	
*We have not taken the role of managing the diseases [HTN and DM] yet, so we refer anyone we discover to the hospital. (Male VHT, Nakaseke town council, VHT 9)* **I don’t diagnose such patients, but if I find them in their homes I urge them to go to a health facility. I do follow up on them. (Female VHT, Nakaseke town council, VHT 11)** *We encourage that they [patients with DM] […] get tested to know their blood sugar levels to ensure that they are well and that it doesn’t continue rising. (Male VHT, Kasangombe, VHT 1)* **[We take care of patients with HTN and DM by] Visiting them and telling them to take their medication. And going back for review to hospital on due day. (Female VHT, Kasangombe, VHT 2)** *I have never been there personally to see how it is done [diagnosis of HTN and DM] but we advise them to go to hospital when they have symptoms. (Female VHT, Nakaseke sub-county, VHT 4)* **We refer patients with DM straight to the hospital when we identify them (Male VHT, Nakaseke sub-county, VHT 6)** *We find these patients and refer them and even do follow up encouraging them to take their medication. (Female VHT, Nakaseke town council, VHT 11)*	
**Concerns about VHT role in HTN and DM care from the perspectives of patients and HCPs**	
*I personally don’t believe that they can do it [manage DM]. It [knowledge on DM] is hard. (HTN and DM, Female, Nakaseke sub-county P7)* **They [VHTs] don’t help us in any way, and they have no role in this disease. I would request that it is the patients that should be trained. […] Their importance is weak, it cannot benefit us in any way. They [VHTs] could be beneficial to other patients such as those with malaria but our disease is so complicated. (DM only, Male, Nakaseke sub-county, P10)** *They [VHTs] have roles in other diseases but not concerning diabetes and hypertension. I do not fully certify them. I don’t think [VHTs can play a role in HTN and DM] maybe malaria and child health. I doubt their abilities. (HTN and DM, Female, Kasangombe, P21)* **I don’t talk with VHTs. They used to treat malaria back then and I have never seen them help patients with hypertension. They are necessary to help children under 5 years old by dispensing malarial drugs but they are not helpful to us the elderly. They do not give out drugs for hypertension and diabetes. They are not trained, they simply help people. The good thing is that we have village clinics even if they [VHTs] are not available. (HTN only, Female, Nakaseke sub-county, P16)**	
*I don’t know their role, but I think HTN and DM are a bit complicated for them. They can only handle the simple diseases like malaria, diarrhea. […] they are trained for HIV but not HTN and DM. And because they have very little knowledge about HTN and DM that most of those people in the villages [with HTN and DM] do not even have relationship with village health team members. They are always trained and given special courses [in HIV], but I never heard any about DM and HTN. (Male, Pharmacist at Nakaseke Hospital HCP6)* **They [VHTs] do a lot of work in the villages, but for HTN and DM, it is above their standard. […] They don’t know enough to convince the patients with HTN and DM. What drug to get, what to eat, and what to drink. (Female, Pharmacist at Nakaseke Hospital, HCP 5)**	

### Patient-reported challenges in HTN and DM care

#### Lack of knowledge of HTN and DM

Patient interviews revealed that patients conceptualized the cause, preventability, treatment, and curability of HTN and DM with limitations. These lay conceptualizations limited patients’ ability to adhere to medications and obtain timely screening. Many participants correctly identified HTN and DM as non-communicable diseases. However, they believed that only communicable diseases were preventable and, therefore, that NCDs were not.

*“They are not communicable like HIV […] You can’t prevent hypertension or diabetes.” (HTN and DM, Female, Kasangombe, P4)*

Some patients described pondering their symptoms for more than a year and delaying hospital screening, only deciding to go to the hospital when their symptoms became severe and affected their daily lives, or after consulting with members of their community. Many patients thought that the cause of HTN and DM was stress, others identified genetics, and some reported a belief that the diseases affected people at random. The belief that stress influenced disease development led some patients to choose relaxation or television watching as HTN and DM self-care. Most patients acknowledged the role of diet and physical activity in HTN and DM management, as informed by their HCPs, but often drew on limited or incorrect information on the topic. Some patients, for example, advocated for avoiding sweet potatoes or soda when feeling weak and one patient reported having been taught what to avoid eating, but never what to eat.

*“You hear them speak of what shouldn’t be eaten but we have not yet gotten a teaching about how to conduct our lives.” (DM only, Female, Kasangombe, P11)*

#### Lay sources of HTN and DM information

Financial constraints and physical distance appeared to be major barriers to accessing health care. Instead of making hospital visits, many patients reported relying on advice from lay sources (family, neighbors, and community) for managing their HTN and DM. The fact that HTN and DM were not associated with any stigma seemed to encourage communication and information sharing about the diseases within the community.

*“Yes, [I feel comfortable sharing with family members and neighbors] because somebody might advise and recommend you to a better medication or to traditional medicines.” (HTN only, Female, Nakaseke Sub-county, P15)*

Most of the information on HTN and DM available to study participants appeared to have been defined collectively by the community, drawing upon experiences of individual community members. As a result, many patients reportedly made decisions to receive triage after their symptoms were legitimized by families and neighbors. Patients also reported sometimes changing their medication (type or dosage) based on community members’ recommendations. While patients reported trusting the information provided by health professionals, open access to lay sources meant they were prone to re-interpret the details such as HTN and DM etiology and curability based on the experiences or beliefs of others in the community.

#### Patient trust in HCPs vs lack of involvement in the medical decision-making processes

Patient interviews revealed that when patients did interact with the healthcare system they had a limited role in the medical decision-making process. Very few patients could name or describe the drugs they were currently taking.

*“Their [the prescribed drugs] names! They [the doctors] just give me the drugs, I don’t know the names. I am just given the drugs.” (HTN and DM, Female, Kasangombe, P4)*

Many patients reported leaving medical decisions completely up to doctors, increasing the medical knowledge gap. This left patients more prone to modifying their treatment without understanding the consequence. As an example, patients would often stop taking their medication or alter its dosage in response to side-effects before consulting with their doctors. Those who changed their medication dosage reportedly did so based on their experience and physical reactions. Some patients who expressed discomfort with the idea of modern drugs also reported terminating medication usage.

*“I get drugs from the hospital so when I have them, I take my drugs. However, I don’t take them daily. I take breaks from them, but there are times when you go back [to the hospital] and if it [the blood pressure] increases, I am given the drugs.” (HTN and DM, Female, Nakaseke-sub county, P6)*

Despite patients’ limited involvement in medical processes, it is important to note that patients did not report dissatisfaction with or concern about the competence of their care. In fact, most patients expressed having trust in their doctors for information and in making medical decisions while they also expressed having trust in lay sources and adherence to traditional herbal medicine.

*“We are in a good relationship with the doctors, because they comfort us and teach us what we need to do to live.” (DM only, Male, Nakaseke sub-county, P8)*

#### Inability to access medication due to financial constraints

Somewhat contradicting their comments on voluntary lack of adherence to medication regimens, patients did uniformly identify drug adherence as the most important treatment for HTN and DM, though also reported that limited access to medication was a major barrier. Although the Ugandan health care system funds most of the health services provided in public hospitals, the hospitals and lower level health centers frequently experience drug shortages. Given these drug shortages at the public pharmacies, patients often are required to purchase medications from private pharmacies, though this is often at a substantial cost. Thus, patients reported financial hardship as an additional barrier to accessing medications due to high costs. As a result, some patients reported reducing their medication dosage or mixing their drugs with traditional herbal medicine. Most patients reported a belief that HTN and DM were curable with consistent adherence to modern drugs. Thus, many patients reported ceasing medication usage once symptoms disappeared.

*“I was told to start on drugs, and to regularly come to [the hospital to] pick up the drugs. However sometimes I go there and the drugs are not available. I also don’t have money. Then I keep quiet with nothing to do.”* (HTN only, Female, Nakaseke town council, P17)

### HCP-reported challenges in HTN and DM care

HCPs frustrations about HTN and DM management largely centered around patients’ lack of adherence to treatment. HCPs reported lack of adherence as resulting from difficulty educating patients or their use of alternative herbal medicines in place of modern medications. They also expressed the belief that the negative reputation of HCPs prevented patients from seeking health care.

#### Negative reputation associated with HCPs

HCPs expressed a frustration with patients’ lack of medication adherence and their subsequently worsening biomarkers and symptoms in follow-up visits. Some HCPs reported that patients feared the healthcare staff because of HCP’s negative attitudes toward patients and their lack of adherence or inconsistency in utilizing medical services.

*“some people fear our attitudes; they don’t want to come to the hospital.” (Female, Nurse at Nakaseke Hospital, HCP1)*

Some of the same HCPs reported that limited drug availability at the hospital and patients’ financial constraints were additional reasons for patients’ failure to follow-up.

#### Patients’ herbal medication use and hospital as the last resort

There were also thoughts from HCPs that patients may not adhere to medications due to a cultural preference for herbal medicine. According to HCPs, most patients with HTN and DM initially draw on herbal medicine and seek out hospital-based treatment as a last resort.

*“people believe in herbal so much even they may not come [to the hospital]. Person comes in too late and say that we’ve been using herbal. […] hospital is always the last solution.” (Female, Nurse at Nakaseke Hospital, HCP3)*

HCPs also expressed concern that patients would sometimes terminate, alter dosage, or take their prescribed medication concurrently with herbal medicine to compensate for missing doses. A few HCPs also expressed concern that taking herbal and modern medicines together might cause antagonistic or negative reactions.

#### Challenges related to patient education on HTN and DM

Interviews with HCPs revealed challenges in their efforts to educate patients with HTN and DM. The reported challenges included lack of resources (educational and human) and time constraints. HCPs reported having no resources, such as clinical or educational guidelines, to use for HTN and DM education. A few HCPs also acknowledged the difficulties of lifestyle education due to existing patient habits, such as routine meat consumption and taking motorized transportation as opposed to exercising. Some HCPs also expressed that the lack of funds and resources to perform community outreach was a barrier to initiating community-based education opportunities.

Additionally, some HCPs reported difficulties in providing HTN and DM education while also serving patients with acute and urgent medical conditions. Some HCPs reported having a lack of staff members with specialized knowledge of HTN and DM. For example, nurses (the only medical professionals running Nakaseke Hospital’s DM clinic) expressed major concerns about not having an HCP with higher-level training present for professional medical assistance. Some HCPs also reported not providing HTN and DM education because of the time commitment required.

*“we’ve not done enough of the counseling, because we don’t have the time […] Diabetes and hypertension needs time [in counseling] because, you need to talk about lifestyles. For both hypertension and diabetes, when we talk about weight reduction, it needs a lot of skills, it needs a lot of examples, needs a lot of talking” (Male, Doctor in Kasangombe, HCP9)*

### VHT role in HTN and DM care

#### Limited scope of VHT responsibilities related to health care

While interviews overall reported VHTs’ active role in communicable disease prevention (exemplified by HCPs indicating that VHTs were especially helpful with regard to HIV management and family planning), VHT interviews revealed their limited role in HTN and DM management. All VHTs defined their current role in HTN and DM as either non-existent or limited to hospital referrals when patients approached them with symptoms.

*“We have not taken the role of managing the diseases [HTN and DM] yet, so we refer anyone we discover to the hospital.” (Male VHT, Nakaseke town council, VHT9)*

#### Concerns about a VHT role in HTN and DM care from the perspectives of patients and HCPs

Both patients and HCPs expressed concern with VHTs taking on roles related to HTN and DM management. Many patients and some HCPs doubted VHT’s ability to understand HTN and DM, voicing the belief that communicable disease prevention is simpler to understand, and NCD prevention, in the case of HTN and DM, too complex.

*“I personally don’t believe that they can do it [manage DM]. It [knowledge of DM] is hard.” (HTN and DM, Female, Nakaseke sub-county, P7)*

*“I don’t know their role, but I think HTN and DM are a bit complicated for them. They can only handle the simple diseases like malaria, diarrhea. […] they are trained for HIV but not HTN and DM. And because they have very little knowledge about HTN and DM that most of those people in the villages [with HTN and DM] do not even have relationship with village health team members. They are always trained and given special courses [in HIV], but I never heard any about DM and HTN.” (Male, Pharmacist at Nakaseke Hospital, HCP6)*

Some HCPs worried that VHT’s might lack motivation to volunteer for additional responsibility related to NCD prevention if VHTs were not compensated monetarily. One HCP cited HIV management as a successful example. She attributed the success of HIV management to organizations that financially supported VHT training, mobilization, and compensation. She expressed her concern about giving VHTs any role in HTN and DM management when there are no such organizations dedicated to HTN and DM in rural Uganda.

## Discussion

Our study provides a closer look at the challenges to HTN and DM care in rural Uganda from the perspective of patients, HCPs, and VHTs. In particular, we found challenges related to patients’ difficulties accessing care and adhering to treatment: patients’ knowledge gaps regarding the preventable aspects of HTN and DM, patients receiving HTN and DM information from lay sources, patients’ mistrust in the Ugandan health care system rather than in individual HCPs, drug shortages due to financial constraints, and patients’ use of herbal medication. Additionally, HCPs report barriers to patient education on HTN and DM, and skepticism was reported from both HCPs and patients regarding a potential role for VHTs in HTN and DM management.

Patients’ limited understanding of the preventable aspects of HTN and DM influenced participants’ health behaviors. While patients with HTN and DM in Nakaseke district correctly identified HTN and DM as NCDs, they were not aware these conditions were preventable based on their non-communicable nature. To our knowledge, this specific assumption formulated by patients has not been observed in previous studies, and therefore may be unique to this region. Previous studies have, however, observed similar patient behavior involving weaving together lay and expert explanations about the causes of illnesses [19]. A study on chronic heart failure patients’ understanding of their illness in Uganda attributed the misconstrued understandings of illnesses mostly to the information lost in translation between doctors and patients [19]. Such misconstrued understandings or inaccurate patient beliefs about the cause of illness are known to lead to late recognition of illness, late seeking of medical care, and changes to where the care is sought [19, 20]. Our finding that patients failed to understand the cause and preventability of HTN and DM implies that there is a need for clear and comprehensive, health literacy-level appropriate health education in Nakaseke and possibly other rural places in Uganda.

Another important finding was that individuals sought information about their health conditions from untrained community members, reporting little fear of stigma associated with revealing their conditions. Patients demonstrated being open to sharing their experiences and knowledge with other members of the community. This lack of stigma is positive for future community-based health promotion efforts. Prior studies in Uganda have also found that patients’ knowledge of NCD and choice of treatments were influenced by community lay sources [19, 20]. Knowledge of treatment options and efficacy has been found to lead patients to seek care more readily [21]. A study found that, with correct information, a community lay health worker (CLHW)-led intervention was effective in promoting medication adherence and reinforcing basic disease education among adults with HTN and/or DM in rural Mexico [22]. CLHWs led patients to promoted positive lifestyle change and improved self-management due to informational and psychosocial supports provided by CHWs [22]. Thus, similar types of interventions may able to produce similar findings among patients with HTN and DM. Community-based health promotion, engaging key community members (such as CLHW or VHTs), may be a novel approach to HTN and DM intervention in this setting.

In prior studies, inadequate communication between providers and patients has frequently been associated with patient dissatisfaction and mistrust of their providers [23, 24], which in turn have been major contributors to patient non-adherence to treatment [24]. While HCPs in this study suggested that similar issues, particularly the patient perception of HCPs as having negative attitudes, were driving the lack of patient follow-up and medication adherence, it is striking that none of the patient interviews indicated this negative perception of HCPs. As such, patients and providers had different perceptions of the challenges in HTN and DM treatment adherence, which speaks to the current level of communication between them. Patient interviews suggested that mistrust of the health care system, rather than mistrust of the HCP personnel, drives patients’ dissatisfaction and impedes their treatment adherence. Based on this finding, joint advocacy efforts by HCP and patients for system change in HTN and DM care, particularly related to medication access, may effectively improve care in rural Uganda.

Patients’ treatment adherence was particularly influenced by drug shortages in rural Uganda. Drug shortages seemed to be the most common reason for patients’ lack of adherence, leading patients to adjust medication dosages. Self-adjustment of medication is a commonly reported coping response in situations where medications are unavailable. For example, a recent study in urban poor settings of South India observed that patients with DM who lacked consistent access to medication altered their prescribed dosage or schedule of intake without seeking medical consultation [25]. The same study also observed some patients compensating for the reduced amount of medication dosage by taking herbal medicine in combination [25].

The use of herbal medication was reported by HCPs to be a major barrier to HTN and DM management. They believed that it was more common for patients to use herbal medication prior to initiating conventional treatments. A study of HTN patients in rural Western Kenya noted that patients took herbal medications in addition to or instead of prescribed medications because of their extensive advertisement, easy availability, and low cost in comparison to conventional medical care [26]. Future research in Uganda should focus on understanding factors associated with herbal medication use and could explore herbal medicine providers as potential partners in care and/or potential sources of referrals to care for patients with HTN or DM.

HCPs also identified time constraints and lack of sufficient healthcare personnel as challenges to educating patients about HTN and DM. This finding of health worker shortages is consistent with previous studies on the state of the health workforce in sub-Saharan Africa (SSA). A previous study reported the proportion of trained doctors and nurses in the region who intend to migrate ranged from 26 to 68% [27]. In an attempt to tackle this issue, studies have explored task shifting specifically in HTN and DM management in LMICs [28, 29]. Task shifting strategies have been seen as a low-cost and applicable action plan that utilizes existing resources such as lower-level health professionals [29]. In our study, however, both patients and HCPs expressed concerns about the ability of VHTs, who would likely take on this role, to cope with prevention and treatment of NCDs.

Much of the skepticism expressed by Nakaseke patients and HCPs toward VHTs’ potential role in HTN and DM care stemmed from their concern that NCD knowledge is different than communicable disease and more difficult to learn. However, recent studies contradict this idea and have shown great potential for VHTs to take part in NCD management [30–32]. For example, a recent study in Eastern Uganda observed that VHTs, despite feeling that they were unprepared for a role in NCD management, already had nuanced knowledge of NCDs and demonstrated willingness be a part of the NCD health care workforce [30]. Another study assessed the potential for expansion of community health workers’ (CHW) role in cardiovascular disease (CVD) management across four LMICs (Bangladesh, Guatemala, Mexico, and South Africa). The study found CHWs to be efficient in screening and monitoring for CVD risk with simplified screening instruments [32]. Furthermore, promising work has been done by the Ministry of Health NCD training program, involving improving NCD knowledge in Ugandan health care workers [33]. While evidence strongly supports that VHTs can indeed be trained to provide HTN and DM education, and we believe that this would be a successful strategy in rural Uganda, additional funding for health finance reform, and administrative support will likely be needed to facilitate this change. Additionally, HCP and community skepticism of the potential for VHT involvement in HTN and DM prevention and care will need to be addressed.

While our study contributed many new insights into the challenges of HTN and DM in rural Uganda, there are several limitations that must be noted. One potential limitation is the gender imbalance in our study sample. While we were able to select close to equal numbers of participants on the axes based on disease and place of residence (or work, for HCP), gender was significantly skewed toward females for patients and HCPs. We initially planned to recruit patients equally by gender, however, more female patients were available and willing to participate than males. Therefore, our findings may represent a limited scope of context that is more relevant to the female patients. Despite the gender imbalance, the HCP sample was actually representative of the care provision in this setting. Secondly, interviewing the three stakeholder groups separately may have not been helpful in reaching consensus regarding the key problems in managing HTN and DM in this setting. For example, only HCPs mentioned fear as a major barrier preventing patients from coming to the hospital. The same idea did not emerge from patients but, rather, patients identified financial constraints and difficulty in accessing facilities as major reasons for their inability to make regular follow-up visits to the hospital. That said, given the power dynamics between the stakeholder groups, participants may have felt less free to share their opinions if they were in mixed groups. Third, as a small regional study, the range of context relevance may be limited and, thus, negatively impact the generalizability of our conclusions. However, we tried to minimize this issue by maximizing selection groups from using axes of diversity on gender, disease, and place of residence. Finally, the list used for initial VHT selection and recommendation could have been biased toward more active VHTs and, thus, the VHT perspectives may not have been reflective of the broader VHT community.

Despite these limitations, our study highlights the idea that there may be challenges to HTN and DM care that are unique to rural Uganda. The perspectives from three key stakeholders gives an overview of the challenges faced by each group. In addition, a major strength of our study is that it allowed us to understand the locally grounded knowledge, attitudes, and practices toward HTN and DM care from the three key stakeholders. Our study findings can contribute to create effective health service interventions that could improve the quality of HTN and DM care in Nakaseke, Uganda.

## Conclusion

Using a qualitative approach, we illuminated the challenges that influenced patients’ compromised access to care and lack of adherence to treatment in rural Uganda. The three major challenges and the novel findings of our study were the following: 1) patients’ knowledge gaps regarding the preventability of HTN and DM, 2) patients’ mistrust in the system of HTN and DM care rather than in HCPs as individuals, and 3) skepticism from HCPs and patients regarding VHTs’ ability to take roles in HTN and DM management. Improving understanding of the preventable aspects of NCDs and addressing systemic issues associated with medication distribution to health facilities are likely to lead to improvements in HTN and DM management in this setting. We recommend implementing a task shifting strategy that utilizes VHTs to educate patients about prevention and cause of non-communicable diseases and expands their existing role to include screening and monitoring of HTN and DM. Based on our findings, HTN and DM education and screening activities conducted on a community level, perhaps engaging community lay health workers, and joint advocacy from HCPs and patients for reforming health systems on medication distribution would be ideal.

## Abbreviations

ACCESS: African Community Center for Social Sustainability
CD: Communicable Disease
CVD: Cardiovascular Disease
DM: Diabetes
FBG: Fasting Blood Glucose level
HCP: Health Care Professional
HIV: Human Immunodeficiency Virus
HTN: Hypertension
IDF: International Diabetes Federation
LMIC: Low-Middle-Income Countries
MOH: Ministry of Health
MREC: Mulago Hospital Research and Ethics Committee
NCD: Non-Communicable Disease
NGO: Non-Governmental Organization
SSA: Sub-Saharan Africa
VHT: Village Health Team member
